# Ibrutinib as a Secondary Treatment for Steroid-Refractory or Steroid-Dependent Chronic Graft-Versus-Host Disease: A Case Series of 11 Patients During the COVID-19 Era

**DOI:** 10.7759/cureus.71474

**Published:** 2024-10-14

**Authors:** Yo Mizutani, Tomoaki Ueda, Jiro Fujita, Kentaro Fukushima, Naoki Hosen

**Affiliations:** 1 Department of Hematology and Oncology, Osaka University Graduate School of Medicine, Suita, JPN

**Keywords:** allogeneic stem cell transplantation, bruton tyrosine kinase inhibitor, chronic graft-versus-host disease, covid-19, ibrutinib

## Abstract

Introduction: Ibrutinib, a Bruton’s tyrosine kinase inhibitor, has recently become available for treating chronic graft-versus-host disease (cGVHD). Because the pivotal clinical trials for this approach were conducted before the COVID-19 pandemic, evidence regarding ibrutinib treatment for cGVHD in the COVID-19 era is insufficient.

Methods: We assessed the safety and efficacy of ibrutinib treatment in a real-world setting by retrospectively analyzing the outcomes of 11 patients with steroid-refractory and steroid-dependent cGVHD who were treated with ibrutinib between November 2021 and April 2024 at our hospital.

Results: The best overall response rate was 63.6%, and the steroid dose was successfully reduced in seven of the patients. Ibrutinib improved hemolytic anemia and serositis in some of the patients. The most common adverse events were infections and bleeding. Four of the patients contracted COVID-19 during the study period, of whom one discontinued the treatment because of severe COVID-19 pneumonia.

Conclusions: Ibrutinib was effective for treating cGVHD, particularly when B cells were involved in its pathogenesis, and decreased the steroid dose needed for treatment in a real-world setting. Patients should be carefully monitored and treated for adverse events during this treatment, particularly bleeding and any complications associated with COVID-19 infection.

## Introduction

Chronic graft-versus-host disease (cGVHD) comprises a serious complication following allogeneic hematopoietic stem cell transplantation (allo-HSCT) that affects 30-70% of patients [1]. It is, therefore, considered a leading cause of morbidity and mortality following allo-HSCT [2,3]. Although steroids are used as a first-line treatment for cGVHD, ~50% of patients become either refractory to or dependent on steroid treatment. Thus, the treatment of steroid-refractory or steroid-dependent cGVHD represents an unmet clinical need. Although a variety of novel therapeutic agents have been introduced as second-line treatments for cGVHD, clinicians should be aware of their variability in terms of efficacy and adverse events [4-6]. Ibrutinib is a first-in-class Bruton tyrosine kinase (BTK) inhibitor that is used to treat B-lymphoid malignancies [7,8]. Its major side effects have been reported to be hypertension, atrial fibrillation, diarrhea, and infections [7,8]. Clinical trials have demonstrated that ibrutinib can effectively treat cGVHD by inhibiting both BTK and interleukin-2-inducible T-cell kinase; however, it remains unclear precisely which types of cGVHD ibrutinib is most effective for treating [9,10]. Certain concerns also exist regarding its impact on infections, particularly COVID-19, owing to its immunosuppressive effects. This study investigated the safety and efficacy of ibrutinib treatment in 11 patients with steroid-refractory or steroid-dependent cGVHD in a real-world setting during the COVID-19 era.

## Materials and methods

Patients and data collection

We retrospectively analyzed the safety and efficacy of ibrutinib treatment in 11 patients with steroid-refractory or steroid-dependent cGVHD who received it as a treatment at Osaka University Hospital (Osaka, Japan) between November 2021 and April 2024. This study complied with the Declaration of Helsinki and was approved by the institutional review board of Osaka University Hospital (approval number: 16265). Written informed consent was obtained from all patients analyzed in this study.

GVHD prophylaxis/treatment and supportive care

In most cases, GVHD prophylaxis consisted of a calcineurin inhibitor plus short-term methotrexate (sMTX). Rabbit anti-human thymocyte globulin or post-transplantation cyclophosphamide (PTCY) was administered to some human leukocyte antigen (HLA)-mismatched patients. Every patient received prednisolone (PSL) as a first-line treatment for cGVHD, and second-line drug selection was at the discretion of each physician. All patients were treated in HEPA-filtered rooms and received fungal, herpes zoster/herpes simplex/cytomegalovirus, bacterial, and *Pneumocystis jirovecii* prophylaxis as well. Although the available treatment modality varied according to the time of year, the main goal was to prevent COVID-19 infection using combinations of drugs.

Definitions

The diagnoses and levels of cGVHD severity were determined based on the 2014 National Institutes of Health (NIH) 2014 Diagnosis and Staging Working Group report [11]. The overall response rate was defined based on the 2005 NIH cGVHD Consensus Panel response criteria [12]. Adverse events were graded according to the National Cancer Institute-Common Terminology Criteria for Adverse Events, version 5.0.

## Results

Patient characteristics

The clinical characteristics of our patient cohort are summarized in Table 1. A total of 11 patients were included (eight men and three women), with a median age of 48 (range, 21-68) years. Their primary diseases were acute lymphoblastic leukemia (five patients), acute myeloid leukemia (three patients), myelodysplastic syndrome (MDS) (one patient), diffuse large B-cell lymphoma (DLBCL) (one patient), and other (one patient). Seven patients received allo-HSCTs from HLA-matched donors, whereas four received them from HLA-mismatched donors. Fungal prophylactic medications were administered, including fluconazole (four patients), voriconazole (two patients), itraconazole (one patient), posaconazole (one patient), and isavuconazole (one patient). Six patients received COVID-19 vaccinations, and four received neutralizing antibodies as prophylactics against COVID-19 infection. Eight patients had a history of acute GVHD, of whom six had undergone grades II-IV acute GVHD.

**Table 1:** Clinical characteristics of patients HLA, human leukocyte antigen; GVHD, graft-versus-host disease; ALL, acute lymphocytic leukemia; AML, acute myelogenous leukemia; MDS, myelodysplastic syndrome; DLBCL, diffuse large B-cell lymphoma; rPBSCT, related peripheral blood stem cell transplantation; uPBSCT, unrelated peripheral blood stem cell transplantation; uBMT, unrelated bone marrow transplantation; FLU, fludarabine; MEL, melphalan; TBI, total body irradiation; AraC, cytarabine, CY, cyclophosphamide; ETP, etposide; Tac, tacrolimus; sMTX, short-term methotrexate; CyA, ciclosporin; ATG, anti-human thymocyte immunoglobulin, rabbit; PTCY, post-transplantation cyclophosphamide; MMF, mycophenolate mofetil

Characteristics	N=11
Median age(range)(years)	48(21-68)
Sex, N(%)	
Female	3(27)
Male	8(73)
Disease, N(%)	
ALL	5(46)
AML	3(27)
MDS	1(9)
DLBCL	1(9)
Other	1(9)
Donor source, N(%)	
rPBSCT	4(36)
uBMT	4(36)
uPBSCT	2(18)
CBT	1(9)
HLA match, N(%)	
match	7(64)
1 allele mismatch	2(18)
2 antigen mismatch	1(9)
haplo	1(9)
Conditioning therapy, N(%)	
FLU/MEL/TBI	4(36)
FLU/BU/TBI	3(27)
FLU/MEL/AraC/TBI	1(9)
FLU/BU/AraC/TBI	1(9)
FLU/TBI	1(9)
CY/ETP/TBI	1(9)
GVHD prophylaxis, N(%)	
Tac+sMTX	7(64)
CyA+sMTX	2(18)
Tac+sMTX+ATG	1(9)
PTCY+Tac+MMF	1(9)
Antifungal prophylaxis, N(%)	
FLCZ	4(36)
VRCZ	2(18)
ITCZ	1(9)
PSCZ	1(9)
ISCZ	1(9)
COVID-19 prophylaxis, N(%)	
Vaccine	6(55)
Neutralizing antibody	4(36)
History of aGVHD, N(%)	
no	3(27)
Grade Ⅰ	2(18)
Grade Ⅱ	5(46)
Grade Ⅲ	1(9)

Clinical outcomes

**Figure 1:**
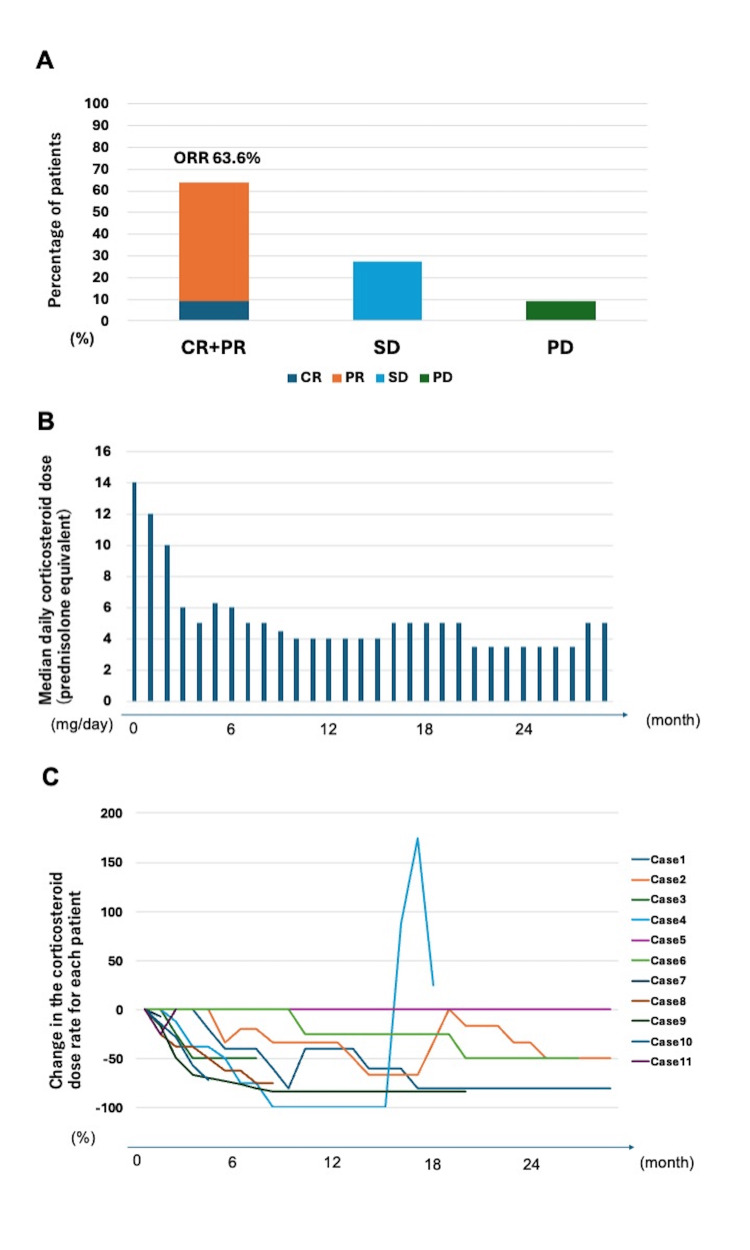
Efficacy of Ibrutinib treatment (n=11) A: Best overall response rate after ibrutinib treatment. B: Median of daily corticosteroid dose (PSL equivalent). C: Changes in corticosteroid doses over time for each patient. CR, complete response; PR, partial response; SD, stable disease; PD, progressive disease; ORR, overall response rate; PSL, prednisolone

The clinical manifestations of cGVHD in the patients are shown in Table 2. All of the patients received PSL as a primary treatment, and six received mycophenolate mofetil (MMF) as a secondary one. The disease severity was severe in nine of the patients, and moderate in two. The affected organs were the skin (seven patients); mouth (five patients); eyes (four patients); liver (two patients); and the gastrointestinal (GI) tract, muscle, pericardium, or others (one patient each). The ibrutinib dose was reduced in ~50% of the cohort because of interactions with other drugs. The median duration of ibrutinib treatment was 607 (range, 17-907) days. Treatment responses included complete response (CR; one case), partial response (PR; six cases), stable disease (SD, three cases), and progressive disease (PD, one case). The median time to best response was 155 (range, 27-642) days. The patients’ response rates are shown in Figure 1A. CR or PR was achieved in seven (63.6%) patients. Two patients were switched to other medications because of poor responses to the treatment. The median daily corticosteroid dose (PSL equivalent) after introducing ibrutinib is shown in Figure 1B. All changes to corticosteroid dosing are shown in Figure 1C. Doses were reduced three and six months after introducing ibrutinib in 37.5% and 45.0% of the patients, respectively. The steroid dose was reduced in seven patients.

**Table 2:** Clinical manifestation of cGVHD NIH, National Institute of Health; cGVHD, chronic graft-versus-host disease; PSL, prednisolone; MMF, mycophenolate mofetil; CR, complete response; PR, partial response; SD, stable disease; PD, progressive disease; GI, gastrointestinal

Characteristics	Total (N=11)
Involved organ, N(%)	
Skin	7(64)
Mouth	5(45)
Eye	4(36)
Liver	2(18)
GI tract	1(9)
Muscle	1(9)
Pericardium	1(9)
Other	1(9)
NIH severity grade, N(%)	
Severe	9(82)
Moderate	2(18)
Prior therapy for cGVHD, N(%)	
PSL + MMF	6(55)
PSL	4(36)
Other	1(9)
Ibrutinib dose, N(%)	
420 mg	4(36)
280 mg	2(18)
140 mg	5(45)
Duration of treatment(range)(days)	607(17-907)
Response	
CR	1(9)
PR	6(55)
SD	3(27)
PD	1(9)
Steroid reduction, N(%)	7(64)
Median time to response(range)(days)	155(27-642)

The results related to drug safety are presented in Table 3. The most frequent adverse event was infection. There were nine cases of infection (COVID-19 in four cases, and invasive pulmonary aspergillosis (IPA), pneumococcal pneumonia, sinusitis, pleurisy, and hemorrhagic cystitis in one case each). Although seven patients received COVID-19 prophylaxis (vaccination in six and neutralizing antibodies in four), four nevertheless developed COVID-19. Two patients discontinued ibrutinib treatment because of severe infection (COVID-19 and pleurisy, one case each). Bleeding was the second most common adverse event, including subcutaneous bleeding (two cases), hematuria (one case), and severe GI bleeding (one case) that led to the discontinuation of ibrutinib.

**Table 3:** Adverse events related to ibrutinib IPA, invasive pulmonary aspergillosis

Adverse events	All grade, N(%)	Grade 3 or higher, N(%)
Patients with one or more adverse events	9(82)	6(55)
Infection	9(82)	6(55)
COVID-19	4(36)	2(18)
IPA	1(9)	1(9)
Pneumococcal pneumonia	1(9)	1(9)
Sinusitis	1(9)	1(9)
Pleurisy	1(9)	1(9)
Hemorrhagic cystitis	1(9)	0
Bleeding	4(36)	1(9)
Subcutaneous bleeding	2(18)	0
Hematuria	1(9)	0
Hematochezia	1(9)	1(9)
Nausea	1(9)	0
Hypertension	1(9)	0

Clinical courses of characteristic cases

**Figure 2:**
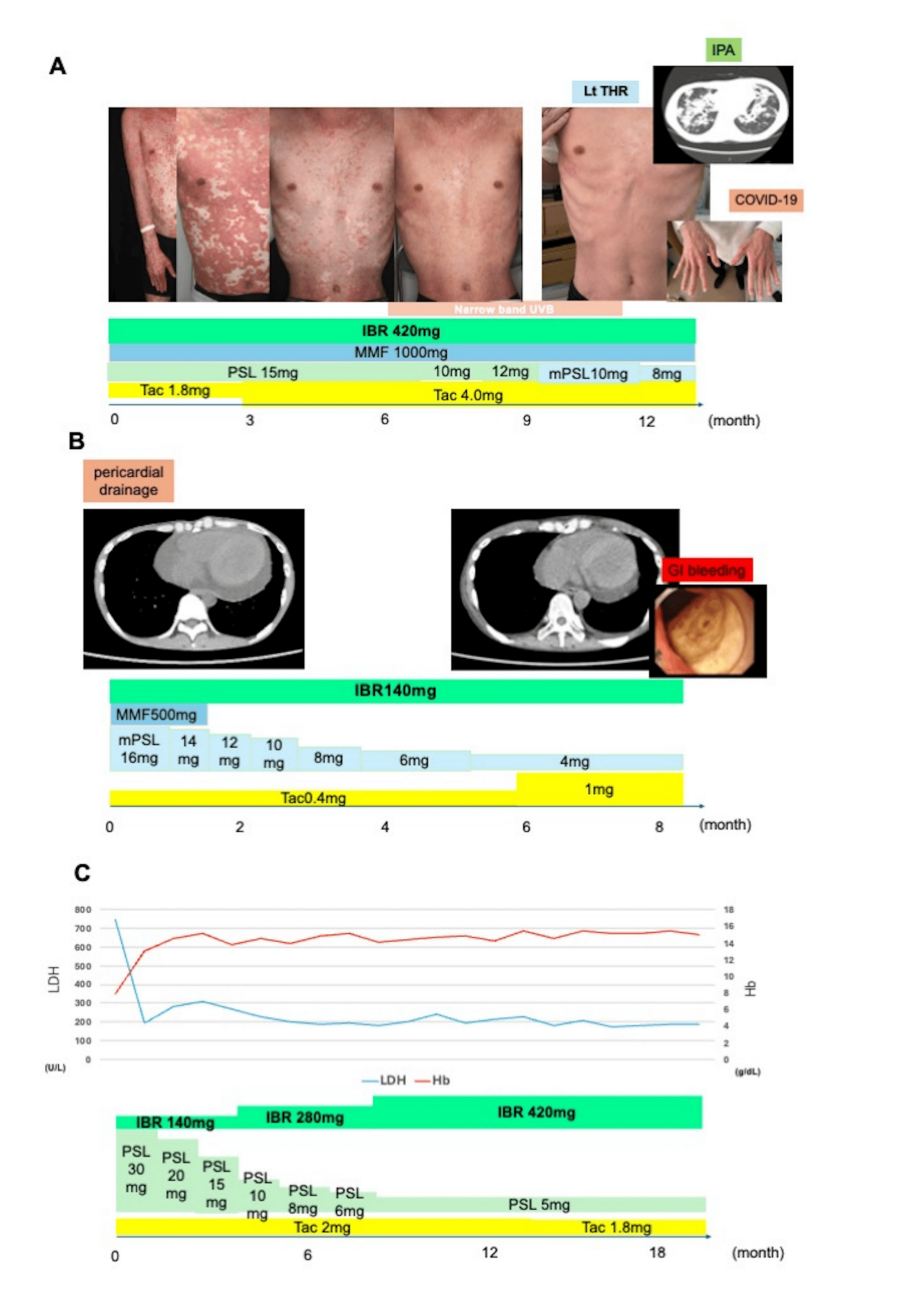
Clinical course of cases 2, 8, and 9 A: Clinical course of case 2. A 25-year-old male underwent uPBSCT from an HLA-matched donor with GVHD prophylaxis of tacrolimus plus sMTX. He developed skin aGVHD, which progressed to steroid-refractory cGVHD. His skin condition gradually improved with ibrutinib and reduced the steroid dose, allowing a left osteotomy for idiopathic osteonecrosis of the femoral head. However, the pt subsequently developed IPA and COVID-19. B: Clinical course of case 8. A 48-year-old female underwent CBT from an HLA 5/8 matched donor with GVHD prophylaxis of tacrolimus plus sMTX. She developed pericardial effusion caused by cGVHD and required pericardial drainage. Ibrutinib improved symptoms and allowed for a reduction in steroid dosage, but it was discontinued due to GI bleeding. C: Clinical course of case 9. A 33-year-old male underwent BMT from an HLA 7/8 matched donor with GVHD prophylaxis of tacrolimus plus sMTX and anti-human thymocyte immunoglobulin, rabbit. He had hemolytic anemia caused by cGVHD and was successfully treated with ibrutinib. The steroid dose was reduced without the recurrence of hemolytic anemia. UVB, ultraviolet B; IBR, ibrutinib; MMF, mycophenolate mofetil; PSL, prednisolone; mPSL, methylprednisolone; Tac, tacrolimus; Lt THR, left total hip replacement; IPA, invasive pulmonary aspergillosis; GI, gastrointestinal; HLA, human leukocyte antigen; sMTX, short-term methotrexate

A 25-year-old male underwent peripheral blood cell transplantation (PBSCT) from an HLA 8/8 matched unrelated donor under a GVHD prophylaxis regimen of tacrolimus plus short-term methotrexate. He developed acute GVHD of the skin, which later progressed to steroid-refractory cGVHD. His skin condition gradually improved within three months after ibrutinib was initiated and resolved almost entirely ~12 months later. The reduction in the steroid dose allowed us to perform a left osteotomy for idiopathic osteonecrosis of the femoral head without any major complications. Although the patient developed IPA and COVID-19, these adverse events were improved with antifungal (liposomal amphotericin B and isavuconazole) and molnupiravir treatments, respectively (Figure 2A). 

A 48-year-old female underwent cord blood cell transplantation from an HLA 5/8 matched unrelated donor under a GVHD prophylaxis regimen of tacrolimus plus short-term methotrexate. She developed pericardial effusion caused by cGVHD and required pericardial drainage. Ibrutinib improved her symptoms and allowed for a reduction in steroid dosage, but was later discontinued because of GI bleeding (Figure 2B). 

A 33-year-old male underwent bone marrow transplantation (uBMT) from an HLA 7/8 matched unrelated donor under a GVHD prophylaxis regimen of tacrolimus plus short-term methotrexate and anti-human rabbit thymocyte immunoglobulin. He had hemolytic anemia caused by cGVHD, which was successfully treated with ibrutinib. The steroid dose was later reduced without recurrence of hemolytic anemia (Figure 2C).

A 54-year-old male underwent PBSCT from an HLA 8/8 matched unrelated donor under a GVHD prophylaxis regimen of tacrolimus plus short-term methotrexate. He developed bronchiolitis obliterans, which was treated using ibrutinib. He later developed COVID-19 pneumonia. Although remdesivir was administered to treat his pneumonia, ibrutinib was discontinued because of his persistent COVID-19 infection and respiratory failure (data not shown).

## Discussion

This study reports a case series of 11 patients with steroid-refractory or steroid-dependent cGHVD who were treated with ibrutinib. To the best of our knowledge, this represents one of the first studies to explore the safety and efficacy of ibrutinib treatment in patients with these conditions in a real-world setting during the COVID-19 era. Seven of the patients (63.6%) achieved CR or PR (Figure 1A), which is consistent with previous studies [9,10]. Steroid doses were successfully reduced in 63.6% of the cases (Figure 1B, 1C). Among these, case 2 achieved the best response (Figure 2A), with complete resolution of planus-like cGVHD and steroid dose reduction. Because of steroid-induced osteonecrosis of the femoral head, the decrease in the patient’s steroid dose was particularly significant. Similarly, the patient in case 8 had serositis and pericarditis-type cGVHD, which were successfully managed using ibrutinib (Figure 2B). Although serositis represents an intractable manifestation of cGVHD, ibrutinib can be used to treat cases of refractory cGVHD with serositis [13]. The patient in case 9 developed hemolytic anemia caused by cGVHD (Figure 2C). Because hemolytic anemia is caused by autoantibodies against erythrocyte membrane antigens, ibrutinib may be more effective than other cGVHD treatments in such cases, as it inhibits antibody production through downregulation of BTK.

Although ibrutinib is effective in patients who are refractory to or dependent on steroid treatment, as well as those who are resistant to conventional therapy, it may cause certain side effects as well. First, ibrutinib increases the risk of infections, including opportunistic ones [14]. In our analysis, four patients contracted COVID-19, of whom one eventually had to discontinue ibrutinib because of severe COVID-19-associated pneumonia. Because ibrutinib may impair serological responses to COVID-19 vaccination, other prophylactic drugs against COVID-19 such as tixagevimab-cligavimab (a neutralizing antibody) should be considered whenever possible, to aid in preventing COVID-19 infections [15,16]. One of our patients contracted IPA (Figure 2A). Appropriate prophylaxis against fungal infections, particularly Aspergillus, should be considered in such cases. Hemorrhage is another relevant side effect that should be carefully managed. Bleeding events occurred in four of our patients, of which one discontinued ibrutinib treatment because of lower gastrointestinal bleeding (Figure 2B). In addition to the inhibition of platelet aggregation by ibrutinib, decreased platelet counts following allo-HSCT may increase the risk of bleeding [17]. Despite the promising efficacy of ibrutinib for managing cGVHD, a significant proportion of the patients in our study discontinued the treatment because of adverse side effects. Given the high rate of discontinuation, it is critical to develop strategies that minimize adverse effects while maintaining the therapeutic efficacy of ibrutinib for cGVHD. Potential approaches to this challenge may include dose adjustments, supportive care interventions, or the use of combination therapies to reduce the dose burden. Careful patient selection and monitoring may help to identify those who are at higher risk of developing severe side effects, allowing for more personalized treatment plans. Another disadvantage of ibrutinib is the slow onset of its therapeutic effects. The median time to best response was 155 days in our cohort, which is consistent with what has been reported previously [9,10]. Therefore, ibrutinib may not be suitable for patients with rapid disease progression.

Several other agents are used as second-line treatments for cGVHD. Ruxolitinib, a Janus Kinase (JAK1/2) inhibitor, has been shown to improve cGVHD [4]. A randomized phase 3 trial reported that ruxolitinib was more effective than ibrutinib for cGVHD; nonetheless, the former caused cytopenia. Because cytopenia is rarely observed during ibrutinib treatment, it is recommended over ruxolitinib in patients with low blood cell counts [8]. Extracorporeal photophoresis (ECP) represents another promising treatment option for cGVHD. Although ECP does not significantly increase the risk of infectious disease and is commonly used as a second-line cGVHD treatment in Western countries, only a few hospitals in Japan offer ECP treatment, because of limited equipment availability. Rho-associated coiled-coil-containing protein kinase-2 inhibitor (belumosudil) is a novel approach to managing cGVHD. This drug regulates Th17/regulatory T-cell balance and profibrotic pathways and has multiple mechanisms of action [6]. Real-world treatment data are anticipated regarding this emerging option. 

This study was subject to certain key limitations worth noting. First, the small sample size prevented us from assessing the effectiveness of ibrutinib in different organs. Owing to the exploratory nature of this study and the small sample size, no control group was used. This limits the statistical power of the findings, making it difficult to draw definitive conclusions. The findings presented here are intended to provide initial insights into the safety and efficacy of Ibrutinib treatment across various pathologies, with the understanding that further studies with larger, controlled samples are warranted for more rigorous statistical analyses. Second, the relatively short observation period in some of our patients limits our ability to assess the long-term safety and efficacy of ibrutinib treatment. Future studies with extended follow-up periods are warranted to provide a more comprehensive understanding of ibrutinib’s role in cGVHD management. Third, since COVID-19 prophylaxis and treatment varied among our patients, the optimal strategies to manage COVID-19 could not be determined. Additional research is warranted to determine the optimal usage of ibrutinib and the most appropriate methods to address COVID-19 in patients with cGVHD.

## Conclusions

Ibrutinib represents the preferred choice for managing cGVHD, particularly when B cells are involved in the pathogenesis. However, as 50% of our patients discontinued ibrutinib treatment because of its side effects, severe adverse events, including COVID-19 infection and bleeding, should be carefully monitored, and appropriate preventive measures should be taken.

## References

[1] Lee SJ (2010). Have we made progress in the management of chronic graft-vs-host disease?. Best Pract Res Clin Haematol.

[2] Arora M, Cutler CS, Jagasia MH (2016). Late acute and chronic Graft-versus-Host Disease after allogeneic hematopoietic Cell Transplantation. Biol Blood Marrow Transplant.

[3] Wingard JR, Majhail NS, Brazauskas R (2011). Long-term survival and late deaths after allogeneic hematopoietic cell transplantation. J Clin Oncol.

[4] Zeiser R, Polverelli N, Ram R (2021). Ruxolitinib for glucocorticoid-refractory chronic graft-versus-host disease. N Engl J Med.

[5] Greinix HT, Ayuk F, Zeiser R (2022). Extracorporeal photopheresis in acute and chronic steroid‐refractory graft-versus-host disease: an evolving treatment landscape. Leukemia.

[6] Jagasia M, Lazaryan A, Bachier CR (2021). ROCK2 inhibition with Belumosudil (KD025) for the treatment of chronic graft-versus-host disease. J Clin Oncol.

[7] Wang ML, Lee H, Chuang H (2016). Ibrutinib in combination with rituximab in relapsed or refractory mantle cell lymphoma: a single-centre, open-label, phase 2 trial. Lancet Oncol.

[8] Woyach JA, Ruppert AS, Heerema NA (2018). Ibrutinib regimens versus chemoimmunotherapy in older patients with untreated CLL. N Engl J Med.

[9] Miklos D, Cutler CS, Arora M (2017). Ibrutinib for chronic graft-versus-host disease after failure of prior therapy. Blood.

[10] Doki N, Toyosaki M, Shiratori S (2021). An open-label, single-arm, multicenter study of ibrutinib in Japanese patients with steroid-dependent/refractory chronic graft-versus-host disease. Transplant Cell Ther.

[11] Jagasia MH, Greinix HT, Arora M (2015). National Institutes of Health consensus development project on criteria for clinical trials in chronic graft-versus-host disease: I. The 2014 diagnosis and staging Working Group report. Biol Blood Marrow Transplant.

[12] Pavletic SZ, Martin P, Lee SJ (2006). Measuring therapeutic response in chronic graft-versus-host disease: National Institutes of Health consensus development project on criteria for clinical trials in chronic graft-versus-host disease: IV. Response criteria working group report. Biol Blood Marrow Transplant.

[13] Modi D, Jang H, Kim S (2016). Incidence, etiology, and outcome of pleural effusions in allogeneic hematopoietic stem cell transplantation. Am J Hematol.

[14] Rogers KA, Mousa L, Zhao Q (2019). Incidence of opportunistic infections during ibrutinib treatment for B-cell malignancies. Leukemia.

[15] Bagacean C, Letestu R, Al-Nawakil C (2022). Humoral response to mRNA anti-COVID-19 vaccines BNT162b2 and mRNA-1273 in patients with chronic lymphocytic leukemia. Blood Adv.

[16] Levin MJ, Ustianowski A, De Wit S (2022). Intramuscular AZD7442 (tixagevimab-cilgavimab) for prevention of Covid-19. N Engl J Med.

[17] Shatzel JJ, Olson SR, Tao DL, McCarty OJ, Danilov AV, DeLoughery TG (2017). Ibrutinib-associated bleeding: pathogenesis, management and risk reduction strategies. J Thromb Haemost.

